# Non-obstructive azoospermia and maturation arrest with complex translocation 46,XY t(9;13;14)(p22;q21.2;p13) is consistent with the Luciani-Guo hypothesis of latent aberrant autosomal regions and infertility

**DOI:** 10.1186/1475-9268-4-2

**Published:** 2005-09-14

**Authors:** Eric Scott Sills, Joseph Jinsuk Kim, Michael A Witt, Gianpiero D Palermo

**Affiliations:** 1 Division of Reproductive Endocrinology and Infertility, Department of Obstetrics and Gynecology, Atlanta Medical Center, Atlanta USA; 2 San Francisco Xavier School of Medicine, Kralendijk, Netherlands Antilles; 3 Reproductive Biology Associates, Atlanta USA; 4 Cornell Institute for Reproductive Medicine, Weill Medical College of Cornell University, New York USA

## Abstract

**Objective:**

To describe clinical and histological features observed in the setting of an unusual complex translocation involving three autosomes (9, 13, and 14) identified in an otherwise healthy male referred for infertility consultation.

**Materials and methods:**

The patient was age 30 and no family history was available (adopted). Total azoospermia was confirmed on multiple semen analyses. Peripheral karyotype showed a 46,XY t(9;13;14)(p22:q21.2;p13) genotype; no Y-chromosome microdeletions were identified. Cystic fibrosis screening was negative. Bilateral testis biopsy revealed uniform maturation arrest and peritubular fibrosis.

**Results:**

Formal genetic counseling was obtained and the extant literature reviewed with the couple. Given the low probability of obtaining sperm on testicular biopsy, as well as the high risk of any retrieved sperm having an unbalanced genetic rearrangement, the couple elected to proceed with fertility treatment using anonymous donor sperm for insemination.

**Conclusion:**

Although genes mapped to the Y-chromosome have been established as critical to normal testicular development and spermatogenesis, certain autosomal genes are now also recognized as important in these processes. Here we present clinical evidence to support the Luciani-Guo hypothesis (first advanced in 1984 and refined in 2002), which predicts severe spermatogenic impairment with aberrations involving chromosomes 9, 13, and/or 14, independent of Y-chromosome status. Additional study including fluorescent in situ hybridization and molecular analysis of specific chromosomal regions is needed to characterize more fully the contribution(s) of these autosomes to male testicular development and spermatogenesis.

## Background

The higher observed frequency of chromosomal abnormality among infertile men compared to the general population provided early evidence that male fertility is influenced by genetic factors impacting gamete development [1]. Many of these genes have been localized to the Y-chromosome, including SRY, AZF, RBM, DAZ, USP9Y, TSPY, DFFRY, and others. A smaller number of genes important to spermatogenesis do not reside on the Y-chromosome [2,3], such as WT1 (chromosome 11), SOX9 (chromosome 17), and DAZLA (chromosome 3), although these autosomal genetic inducers/regulators of gamete development remain less understood. Both Luciani *et al *[4] and Guo *et al *[5] have suggested roles for several autosomes in spermatogenesis, although their hypothesis has proven difficult to test. In this report, we present an unusual complex translocation involving portions of chromosomes 9, 13, and 14 associated with azoospermia and discuss its relevance to the Luciani-Guo hypothesis.

## Case report

**Figure 1:**
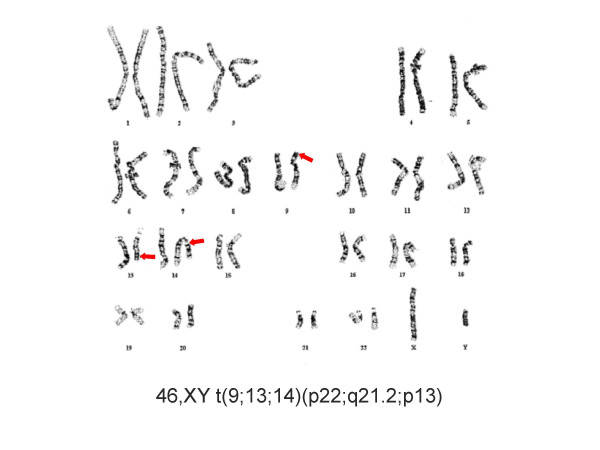
Peripheral karyotype of a male with azoospermia. Complex translocation of chromosomes 9, 13, and 14 was observed; Y-chromosome microdeletion analysis was normal.

**Figure 2:**
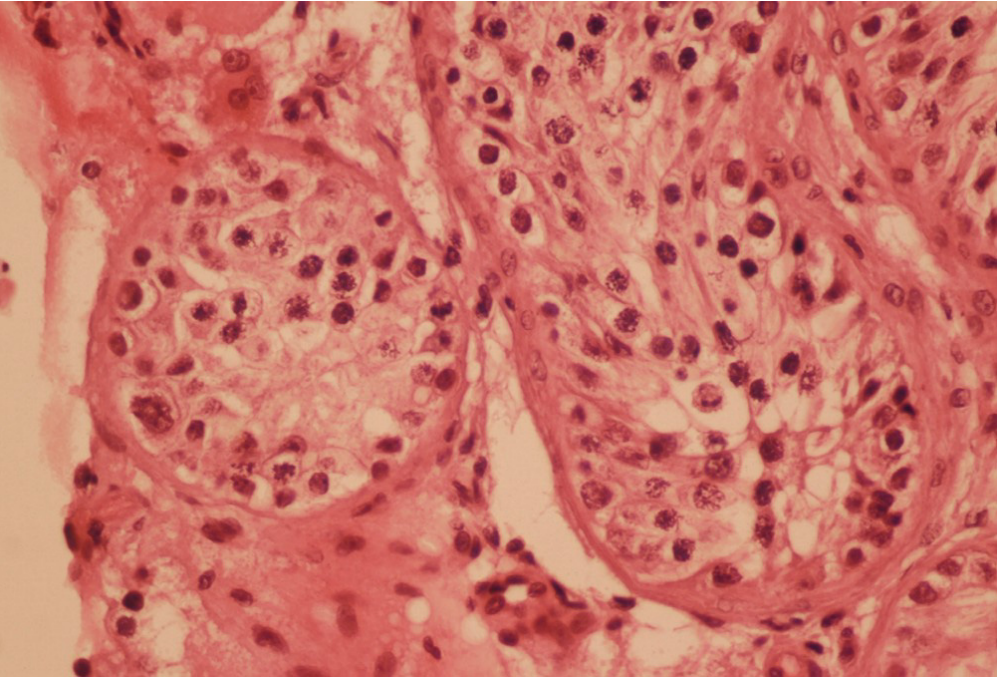
Testicular histology observed in 46,XY t(9;13;14)(p22;q21.2;p13) patient. Seminiferous tubules containing only early germ cell forms were noted, but no fully developed sperm (maturation arrest). Peritubular fibrosis and scattered collections of Leydig cells were also present within the interstitium.

A healthy Caucasian couple was referred for reproductive endocrinology consultation after attempting to achieve pregnancy for >1 yr. The female was age 33 and her evaluation was entirely normal. The husband's semen analysis showed total azoospermia and this was verified on subsequent studies. Cystic fibrosis testing showed no mutation and no Y-chromosome microdeletions were identified (20 cell analysis). Serum testosterone was 415 ng/dl; prolactin was also normal at 6.7 ng/ml. Serum LH and FSH were 2.4 and 2.6 mIU/ml, respectively. Peripheral blood karyotype of 20 cells revealed a non-mosaic 46,XY t(9;13;14)(p22;q21.2;p13) result [Fig. 1]. The patient was adopted and therefore family history was noninformative. Reproductive urology evaluation was sought and outpatient bilateral testis biopsy proceeded without complication. Testicular histology showed peritubular fibrosis and a uniform pattern of Leydig cell hyperplasia with spermatogenic maturation arrest [Fig. 2].

Genetic counseling was engaged and the couple was informed about the exceedingly rare nature of the husband's chromosomal aberration. Mathematical modeling to predict possible permutations for gamete recombination [6] confirmed that even if sperm were recovered via testicular biopsy for use with intracytoplasmic sperm injection, the risk for abnormal offspring was unacceptable. Based on these considerations, the couple elected to undergo ovulation induction and intrauterine insemination using anonymous donor sperm.

## Discussion

Initial generalizations that male gamete organization and function were directed exclusively by genes on the Y-chromosome required revision when it was observed that certain autosomal anomalies caused gonadal failure, even when the Y-chromosome was intact. In 1984 Luciani *et al *described a male with non-obstructive azoospermia having a 13q;14q translocation in the absence of any Y-chromosome aberration. From this it was hypothesized that regions specifically involving chromosomes 13 and/or 14 might be required for normal testicular development [4]. A later investigation by Guo *et al *[5] studied >100 infertile Chinese males and extended the roster of candidate autosomes to include chromosomes 9 and 21. While our patient did not manifest an abnormality of chromosome 21, for many years absent spermatogenesis was considered typical for males with non-mosaic trisomy 21. Additional investigation demonstrated this was not always the case, however [7]. From this it may be concluded that some autosomes are more critical to gonadal structure and/or function than others.

Complex translocations involving more than two chromosomes are highly unusual events in humans [8]. In some carriers of chromosomal aberrations resulting in pachytene configurations with terminal asynaptic autosomal segments, there is a gradual association of asynaptic segments with the X-Y body [9]. Meiotic studies have suggested disordered spermatogenesis may result from such abnormal chromosome synapsis, as any condition interfering with X-Y bivalent formation or X-chromosome inactivation is critical in meiosis. Indeed, the asynapsed regions themselves may trigger developmental arrest of spermatocytes harboring meiotic error [1]. Since simple reciprocal translocations affecting just two chromosomes have deleterious effects on male fertility, it is not surprising that complex translocations involving three or more chromosomes would have drastic and severe consequences for gametogenesis. Even if fertility were maintained for such patients, the risk of genetic error in the offspring would be unacceptably high for most patients [10].

An ideal opportunity to test the Luciani-Guo hypothesis regarding chromosomes 9, 13, and 14 would present whenever these rearrangements occur, although until now there has been no description of rearrangements involving more than two of the implicated autosomes in the same individual. As a further complicating matter, tissue from gonadal biopsy is not always present to complete the evaluation.

This is the first published case of complex translocation involving chromosomes 9, 13, and 14. In association with this exceptional genetic rearrangement, our patient was azoospermic and testis biopsy showed maturation arrest. After a formal consultation with a geneticist, the clinical significance of this genotype was discussed and previous reports describing similar chromosomal abnormalities were reviewed. Based on these data the couple elected to pursue ovulation induction and intrauterine insemination with anonymous donor sperm. While relationships between specific chromosome aberrations and disease conditions should be confirmed by additional study, our data suggest that at present males with this particular complex translocation are poor candidates for surgically retrieved sperm for use with intracytoplasmic sperm injection, since the risk of obtaining genetically abnormal sperm from such individuals is high.

## References

[B1] Antonelli A, Gandini L, Petrinelli P, Marcucci L, Elli R, Lombardo F, Dondero F, Lenzi A (2000). Chromosomal alterations and male infertility. J Endocrinol Invest.

[B2] Okabe M, Ikawa M, Ashkenas J (1998). Male infertility and the genetics of spermatogenesis. Am J Hum Genet.

[B3] Kent-First M, Muallem A, Shultz J, Pryor J, Roberts K, Nolten W, Meisner L, Chandley A, Gouchy G, Jorgensen L, Havighurst T, Grosch J (1999). Defining regions of the Y-chromosome responsible for male infertility and identification of a fourth AZF region (AZFd) by Y-chromosome microdeletion detection. Mol Reprod Dev.

[B4] Luciani JM, Guichaoua MR, Mattei A, Morazzani MR (1984). Pachytene analysis of a man with a 13q;14q translocation and infertility. Behavior of the trivalent and nonrandom association with the sex vesicle. Cytogenet Cell Genet.

[B5] Guo J-H, Zhu P-Y, Huang Y-F, Yu L (2002). Autosomal aberrations associated with testicular dysgenesis or spermatogenic arrest in Chinese patients. Asian J Androl.

[B6] Benito C, Gallego A (2004). A simple method for the estimation of recombination frequencies and genetic distances. Cell Mol Biol Lett.

[B7] Sheridan R, Llerena J, Matkins S, Debenham P, Cawood A, Bobrow M (1989). Fertility in a male with trisomy 21. J Med Genet.

[B8] Siffroi JP, Benzacken B, Straub B, Le Bourhis C, North MO, Curotti G, Bellec V, Alvarez S, Dadoune JP (1997). Assisted reproductive technology and complex chromosomal rearrangements: the limits of ICSI. Mol Hum Reprod.

[B9] Solari AJ (1999). Synaptonemal complex analysis in human male infertility. Eur J Histochem.

[B10] Grasshoff U, Singer S, Liehr T, Starke H, Fode B, Schoning M, Dufke A (2003). A complex chromosomal rearrangement with a translocation 4;10;14 in a fertile male carrier ascertainment through an offspring with partial trisomy 14q13→q24.1 and partial monosomy 4q27→q28 [corrected]. Cytogenet Genome Res.

